# Statistical Optimization of Biosurfactant Production from *Aspergillus niger* SA1 Fermentation Process and Mathematical Modeling

**DOI:** 10.4014/jmb.2303.03005

**Published:** 2023-06-09

**Authors:** Mansour A. Al-hazmi, Tarek A. A. Moussa, Nuha M. Alhazmi

**Affiliations:** 1 Department of Biological Sciences, Faculty of Sciences, King Abdulaziz University, P.O. Box 80200, Jeddah 21589, Saudi Arabia; 2 Botany and Microbiology Department, Faculty of Science, Cairo University, Giza 12613, Egypt; 3 Department of Biology, College of Science, University of Jeddah, Jeddah 21589, Saudi Arabia

**Keywords:** Biosurfactant, statistical optimization, *A. niger*, mathematical model, submerged fermentation

## Abstract

In this study, we sought to investigate the production and optimization of biosurfactants by soil fungi isolated from petroleum oil-contaminated soil in Saudi Arabia. Forty-four fungal isolates were isolated from ten petroleum oil-contaminated soil samples. All isolates were identified using the internal transcribed spacer (ITS) region, and biosurfactant screening showed that thirty-nine of the isolates were positive. *Aspergillus niger* SA1 was the highest biosurfactant producer, demonstrating surface tension, drop collapsing, oil displacement, and an emulsification index (E_24_) of 35.8 mN/m, 0.55 cm, 6.7 cm, and 70%, respectively. This isolate was therefore selected for biosurfactant optimization using the Fit Group model. The biosurfactant yield was increased 1.22 times higher than in the nonoptimized medium (8.02 g/l) under conditions of pH 6, temperature 35°C, waste frying oil (5.5 g), agitation rate of 200 rpm, and an incubation period of 7 days. Model significance and fitness analysis had an RMSE score of 0.852 and a *p*-value of 0.0016. The biosurfactant activities were surface tension (35.8 mN/m), drop collapsing (0.7 cm), oil displacement (4.5 cm), and E_24_ (65.0%). The time course of biosurfactant production was a growth-associated phase. The main outputs of the mathematical model for biomass yield were Yx/s (1.18), and μ_max_ (0.0306) for biosurfactant yield was Y_p/s_ (1.87) and Y_p/x_ (2.51); for waste frying oil consumption the S_o_ was 55 g/l, and K_e_ was 2.56. To verify the model’s accuracy, percentage errors between biomass and biosurfactant yields were determined by experimental work and calculated using model equations. The average error of biomass yield was 2.68%, and the average error percentage of biosurfactant yield was 3.39%.

## Introduction

Chemical surfactants are considered an environmental hazard due to the difficulty of their degradation. Still, these compounds are among the most versatile materials in the chemical and process industry. Their amphiphilic nature—the fact that they contain both hydrophilic and lipophilic functional groups in one molecule—plays an important role in numerous chemical applications (dispersion systems, such as emulsions and colloids, personal hygiene, detergents, fabric softeners, emulsions, paints, and food additives) [1-3].

The primary physicochemical function of surfactants is to lower surface and interfacial tension at immiscible liquid, solid, and gas interfaces, enabling different phases to mix and cooperate [4, 5]. They participate in a wide range of industrial market categories, including those where products are currently in demand as a result of the COVID-19 pandemic [6, 7]. Numerous items, including toothpaste, soap, detergents, fabric softeners, etc., contain a substantial amount of surfactants [5]. The majority of chemical surfactants are made from petrochemicals, which, despite being commercially feasible, are environmentally unfavorable [4, 5]. The development of safer and more environmentally friendly industrial bioprocesses, preferably utilizing ecological biomolecules with superior structural and functional features, is a continual endeavor for biotechnology and chemical firms [8].

Although filamentous fungi have also been shown in multiple studies to create biosurfactants, bacteria and yeast still produce the majority of them [9-11]. These microorganisms are primarily isolated from soil, and they have the potential to create a wide range of substances for biotechnological uses, including enzymes, pigments, and antibiotics [12]. The kind and concentration of the carbon source, the concentrations of other nutrients including nitrogen, magnesium, phosphorus, iron, manganese, and sulfur, as well as the pH, agitation, temperature, and oxygen, all have an impact on the formation of biosurfactants by microorganisms [3, 13].

In general, fungal biosurfactants have versatile chemical structures and their properties allow a wide range of applications. These include lubrication, detergency, emulsification, foaming capacity, production ability, dispersion phases and solubility [3, 14-16], biosensing, catalysis, electronics [17], the personal care [18] and food industries [19], agriculture [20], pharmaceuticals [21], biomedicine [22], materials engineering [23], bio-energy [24], and environmental remediation [25, 26].

The advantages of biosurfactants (surfactants of biological origin) by comparison with surfactants from the chemical industry (*i.e.*, lower toxicity, higher biodegradability, and possible biological activities) have been discussed in diverse types of literature [3, 14, 27].

In this research, we present concepts on the thermodynamic and physicochemical characteristics of biosurfactants to allow for a thorough investigation of their composition and use. We also demonstrate promising niches for biosurfactant-producing fungus isolation and discuss screening approaches. Lastly, related methods including process variables and parameters, concurrent manufacturing, and process optimization using statistical tools are covered as well.

## Materials and Methods

### Sample Collection

Ten oil-polluted soil samples were collected from old, out-of-service fuel stations in Khulais Governorate, Makkah Province, Saudi Arabia. The soil samples were collected in clean, sterilized Falcon tubes (50 ml). The coordinates of the sites were presented in Table S1.

### Isolation and Spore Density of Fungal Strains in the Soil

For direct isolation of fungi, two selective media were used. The first, Erythritol-Chloramphenicol Agar (ECA), was prepared with Yeast-Nitrogen-Base medium (Difco, USA) with 1% meso-erythritol (1,2,3,4-butantetrol; Roth, Germany), 0.05% chloramphenicol (Merck, Germany) and 2.5% agar (UiPath, Germany). Incubation was at 30°C, which is selective for black yeast spp. and some other species [28]. The second selective medium was a mixture of the Peptone PCNB Agar [29] and the PCNB medium with Rose Bengal described by [30], which is selective for *Fusarium* spp. and relatives. The composition of the medium was: glycerin 10 g, urea 1 g, L-alanine 0.5 g, PCNB 1 g, Rose Bengal 0.5 g, agar 15 g, and water 1,000 cm^3^. The ingredients were mixed and autoclaved at 121°C for 20 min and 50 mg/dm^3^ streptomycin was added after autoclaving.

### Extraction of DNA and PCR

For molecular identification, the forty-four fungal isolates were typed via DNA isolation and sequencing of the ITS region. DNA extraction was performed from 0.5 g fungal mycelium collected after 5 days of incubation using Mixer Mill isolation protocol and the polymerase chain reaction (PCR) was run in triplicate. The reaction was carried out in a 25 μl volume containing 1× PCR buffer, 1.5 mM MgSO_4_, 2mM dNTP mixture, 1 μM of each primer, 1 μl of *Pfu* DNA polymerase (Fermentas, St. Leon-Rot, Germany) and 1 ng of template DNA. PCR amplification was performed as follows: initial denaturation at 95°C for 5 min, followed by 25 cycles each of 94°C for 1 min, 55°C of annealing for 45 s, and a 45 s extension at 72°C. The PCR products amplified from the fungal isolates which appeared as a single band were purified and sequenced. All fungal isolates were then checked with the GenBank sequence databases. All obtained sequences were aligned and analyzed in MEGA5.

### Dye-Binding Assay for Screening of Biosurfactant Production

The ability of the forty-four fungal isolates to create biosurfactants was tested. To assess dye-binding activity, the modified CTAB-MB agar medium was employed [31]. The development of a blue ring around the colony served as a sign that biosurfactant was being produced.

### Biosurfactant Production

For liquid fermentation, 10^5^ spores/ml was inoculated into a 500-ml flask containing 150 ml fungal growth medium consisting of (g/l): NaNO_3_, 3; KH_2_PO_4_, 1.0; yeast extract, 1.0; peptone, 3.0; and MgSO_4_·7H_2_O, 0.5. The medium was generally amended with 5 g/l waste frying oil as a substrate. The culture temperature and agitation rate were 30°C and 200 rpm, respectively. The pH of the medium was initially adjusted to 6.8 by 1.0 M HCl.

The samples were centrifuged at 3,000 g for 3 min to remove biomass. Floating biomass that was difficult to sediment was filtered using a 0.24 μm membrane filter (Millex Millipore, USA). The filtrate was used for surface activity tests as described below.

### Biosurfactant Characterization

To evaluate the most potent fungal isolates for biosurfactant production, the culture filtrate was measured using surface tension, oil displacement, drop collapsing, and emulsification index (E_24_).

### Surface Tension

To measure surface tension using the du Nouy ring type method on a tensiometer (Lauda-Königshofen, Germany), the control was set on the uninoculated medium, and calibration was performed using ethanol and pure water. After carefully dipping the platinum ring until equilibrium was attained, all measurements were carried out at room temperature [32].

### Oil Displacement

The oil displacement test was used with minor modifications for the determination of biosurfactant production by fungal isolates. The used engine oil (100 μl) was placed on the surface of the Petri dishes containing distilled water (40 ml), then cell-free broth (10 μl) was dropped on the oil-coated thin film, and the diameter of the circle clear zone of displaced oil was measured [33].

### Drop Collapsing

Using crude oil as the hydrocarbon substrate, the drop collapse assay was carried out using a slightly modified Bodour and Miller-Maier method [34]. One drop of crude oil was placed on a glass slide, and then one drop of culture broth that had been developed for 48 h was dropped onto the crude oil drop. Drop collapse activity was then seen to occur.

### Emulsification Index (E_24_)

The emulsifying capacity was evaluated by an emulsification index (E_24_). The E_24_ of culture samples was determined by adding 2 ml of oil and 2 ml of the cell-free broth to a test tube, vortexed at high speed for 2 min, and allowed to stand for 24 h. The E_24_ index is given as the percentage of the height of emulsified layer (cm) divided by the total height of the liquid column (cm). The emulsification index percentage was calculated using the following equation [35, 36]:

$${\text{Emulsification index (E}}_{\text{24}}\text{)=}\left(\frac{\text{Hight of the emulsion formed (cm)}}{\text{Total hight of the solution (cm)}}\right)\text{x100.}$$

### Optimization of the Cultural Conditions for Biosurfactant Production

The design of experiment (DOE) was carried out using JMP^©^ statistical software [37]. A large number of experimental situations are described as DOEs were used to reduce experimental errors and enhance the efficiency and reproducibility of the laboratory experiments. Five factors (pH, temperature, waste frying oil, agitation rate, and inoculum size) that showed significant influence on biosurfactant production [38, 39] were considered in the present experimental situation (Tables S2a and S2b).

### Time Course of Biosurfactant Production by *A. niger* SA1

*A. niger* SA1 was cultivated on the optimized medium and at time intervals (3, 5, 7, 9, 11 and 13 days), the biosurfactant production, biomass yield, and substrate residual (waste frying oil) were measured.

### Mathematical Model of Biosurfactant and Biomass Production, and Oil Consumption

The logistic model is able to describe the rate of biomass change as a function of biomass alone in both exponential and stationary phases; the relationship can be explained through the following equation:

$$\frac{dx}{dt}=\mu_{\max}X\left(1-\frac{X}{X_{\max}}\right),$$ (1)

which on integration, with the initial condition that at t=0, X=Xo, yields

$$\ln\frac{X}{\left(X_{\max}-X\right)}=\mu_{\max}t+\ln\frac{X_{0}}{X_{\max}-X_{0}}$$ (2)

where X is the biomass concentration at time (t), X_o_ is the initial biomass concentration (inoculum), X_max_ is the maximum biomass concentration and μ_max_ is the maximum specific growth rate.

On rearrangement, and explicit function for biomass is obtained as:


$$X=\frac{X_{0}e^{\left(\mu_{\max}{}^{t}\right)}}{1-\frac{X_{0}}{X_{\max}}(1-e^{\left(\mu_{\max}{}^{t}\right)}}$$ (3)

Biosurfactant balance yields (P) and maintenance coefficient as shown in Eq. (4):

$$\frac{ds}{dt}=\frac{1}{Y_{x/s}}x\frac{dX}{dt}+\frac{1}{Y_{p/s}}x\frac{dP}{dt}+K_{e}X,$$ (4)

where Y_x/s_ is the biomass concentration coefficient, Y_p/s_ is the biosurfactant concentration coefficient, K_e_ is the maintenance coefficient, and dP/dt is biosurfactant production.

The last term in Eq. (4) including the maintenance coefficient accounts for the oil consumption for fungal cell sustainability, viability, and other activities such as enzyme production, osmotic regulation, assimilation, and nutrient storage.

The model of the biosurfactant production in terms of the relationship with fungal growth can be described using Gaden’s classification. This relationship is classified into three different classes: in class I, product formation is connected to fungal growth; in class II, product formation is partially connected to fungal growth, and in class III, product formation is unrelated to fungal growth.

So, in general the biosurfactant formation rate can be expressed as shown in Eq. (5):

$$\frac{dP}{dt}=\alpha \frac{dX}{dt}+\beta x,$$ (5)

where α is the biosurfactant production coefficient and β is the nongrowth correlation coefficient.

The biosurfactant production by *A. niger* is the first class which means α ≠ 0 and β = 0.

$$\frac{dP}{dt}=\alpha \frac{dX}{dt}$$ (6)

By integration:

P=αX+K, (7)

where K is the constant of integration.

Eq. (4) can be simplified by using Eq. (6) to production Eq. (8)

$$\frac{dS}{dt}=\frac{dX}{dt}\left(\frac{1}{Y_{\frac{x}{s}}}+\frac{\alpha }{Y_{\frac{p}{s}}}\right)+K_{e}X$$ (8)

After substituting dX/dt by Eq. (3) in Eq. (8), and by integration, the substrate (oil) concentration was obtained from the following expression:

$$S=S_{0}\frac{X_{0}X_{\max}e^{\left(\mu_{\max}{}^{t}\right)}}{Y_{x/s}(X_{\max}-X_{0}+X_{0}X_{\max}e^{\mu_{\max}{}^{t}})}+\frac{X_{0}}{Y_{x/s}}-\frac{X_{\max}K_{e}}{\mu_{\max}}\ln\left(\frac{X_{\max}-X_{0}+X_{\max}X_{0}e^{\left(\mu_{\max}{}^{t}\right)}}{X_{\max}}\right),$$ (9)

where S_o_ is the initial substrate (waste frying oil) concentration.

The yield coefficient for the biosurfactant may be deduced from Eqs. (7) and (9) giving:

$$Y_{p/s}=\alpha Y_{x/s}$$ (10)

## Results

### Isolation, Spore Density, and Identification of Fungal Strains in the Soil

The spore density of the fungi detected in the ten soil samples revealed that soil sample (1) was the highest soil sample in fungal spores with 18 colonies, followed by soil sample (2) with 13 colonies, and soil sample (10) with 5 colonies (Fig. S1). *Aspergillus niger* was the most dominant fungal isolate when the ten soil samples were cultivated on the two media (ECA and PCNB), followed by *A. terreus* and *A. egypticus*. The ECA medium gave a higher diversity of fungi than the PCNS medium (Figs. S2 and S3). Most strains proved to belong to the genus *Aspergillus* as predicted (Figs. S2 and S3 for the different media).

**Fig. 1:**
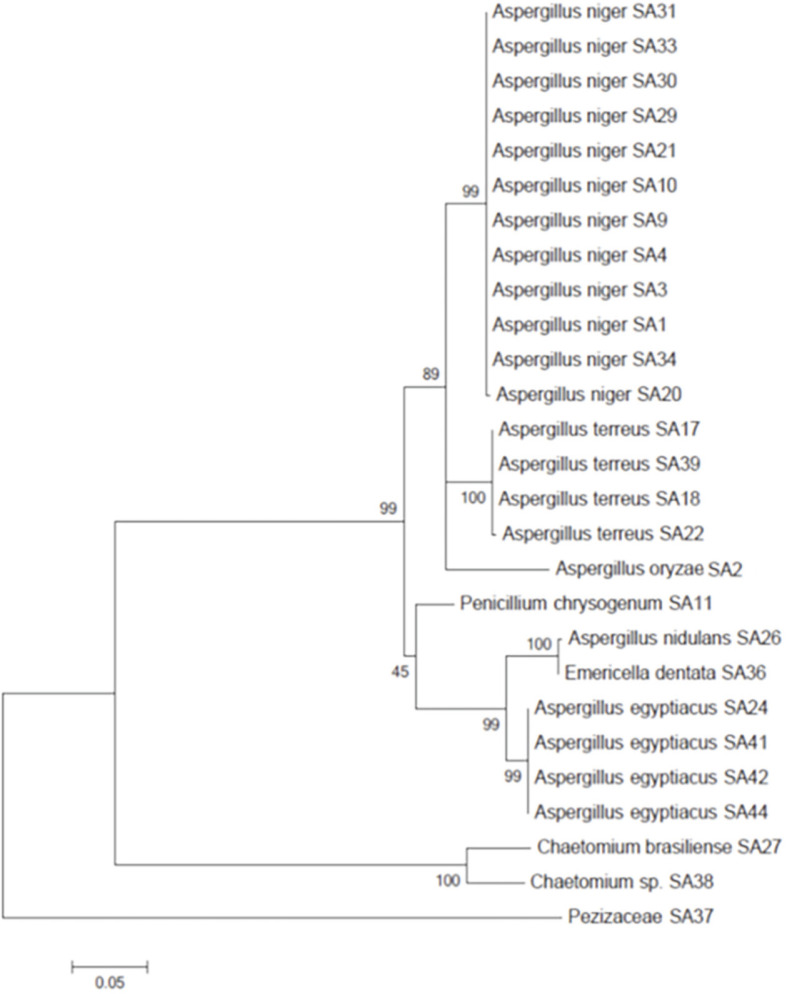
Maximum likelihood tree based on the ITS sequences of all fungal isolates. At the nodes, 1000 bootstrap values are given. The tree was rooted with the unidentifiable *Pezizomycetes* sp. SA37 (Mega 5).

The colonies on both media were further typed via DNA isolation and sequencing of the ITS region. All strains were then checked with the GenBank/CBS sequence databases. All obtained sequences were aligned and analyzed in MEGA5. The best-fitting substitution model for the alignment proved to be Kimura 2 parameter model with gamma distribution. A maximum-likelihood tree was constructed based on the ITS sequence of all strains with 1,000 bootstrap values given at the nodes (Fig. 1).

### Screening of Biosurfactant Production by the Isolated Fungi

All fungal isolates were screened to produce biosurfactant by using the halo zone technique (Table 1). All forty-four fungal isolates were capable of biosurfactant production except *Aspergillus nidulans*, *Chaetomium brasiliense*, *Chaetomium* sp., and *Emericella dentata*. The highest zone diameter was obtained with *A. niger*, followed by *A. flavus* and *Penicillium chrysogenium*. The five, highest-producing fungal isolates were selected for biosurfactant production and measurements of surface activity parameters.

The production of biosurfactant from *A. niger* SA1 was the highest confirming the results obtained from the halo zone. The surface tension, drop collapsing, oil displacement and emulsification for the biosurfactant produced by *A. niger* SA1 were 35.8 mN/m, 0.55 cm, 6.7 cm and 70%, respectively (Table 2). This fungal strain was selected for further optimization and cultural kinetics.

### Optimization of the Cultural Conditions for Biosurfactant Production

The factors used to build up the model and the responses were summarized in Tables S2a and S2b. The initial model used in designing the experiment included the following model terms: X1, X2, X3, X4, X5, X1*X2, X1*X3, X2*X3, X1*X4, X2*X4, X3*X4, X1*X5, X2*X5, X3*X5, X4*X5, X1*X1, X2*X2, X3*X3, X4*X4, X5*X5.

**Fig. 2:**
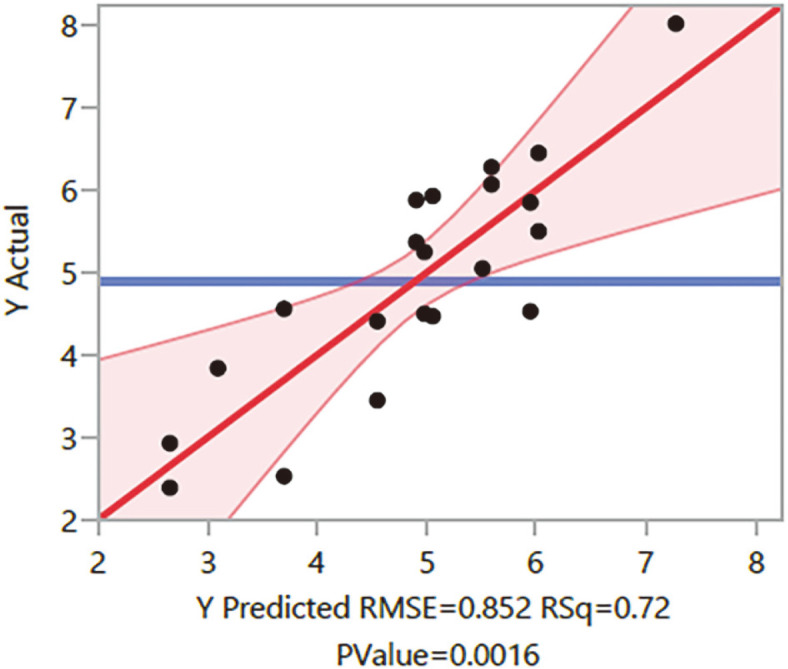
Response means the actual by the predicted plot of the biosurfactant production. Mean Predicted RMSE.

The Fit Group model was generated using JMP software to optimize the production of biosurfactant from *A. niger*, SA1, and 5 factors with three levels each which required 23 experiments, and the response (biosurfactant yield (g/l)) were determined (Table 3). The biosurfactant yield was in the range of 2.39-8.02 g/l depending on the cultural conditions. The best culture conditions for the highest production of biosurfactant (8.02 g) were pH 6, temperature 35°C, waste frying oil 5.5 g, agitation rate 200 rpm, and an incubation period of 7 days. In other experimental runs, the biosurfactant yield varied with the conditions. The lowest biosurfactant yield was obtained at the culture conditions of pH 7, temperature 25°C, waste frying oil 6.5 g, agitation rate 150 rpm, and an incubation period of 5 days. The model significance and fitness were analyzed by the determination of root mean square error (RMSE = 0.852) and *p*-value (0.0016) through the actual by predicted plot (Fig. 2). A red 95%confidence region is plotted on top of the slanted red line. The horizontal blue line is not contained within the red region, so the whole model test is significant at the alpha = 0.05 level.

The plot can also be used to visually evaluate the possibility of a 'lack of fit.' An unbiased prediction should produce predicted values that agree with the observed values on average. The red line went through the middle of the data points.

The most effective factors in the production of biosurfactant were waste frying oil concentrations, agitation rate, temperature, and incubation time, while the least effective factor was pH value (Table S3). These were detected in the FDR log worth where the combination of X2 and X4 was the most significant factor (2.174) and *p*-value 0.00671, followed by X4 (FDR log worth 2.037 and *p*-value 0.00919) and X5 (FDR log worth 1.697 and *p*-value 0.02009).

The model was analyzed by the determination of ANOVA variant and each factor’s combinations were tested using F ratio, t ratio, Prob > |t|, and Prob > F (Tables 4 and 5). Final parameter estimates for the remaining terms after model selection were analyzed, where the intercept had the highest estimate and t ratio (5.0552 and 12.46, respectively). The highest combinations were X2*X4 (temperature*agitation rate) with estimate and t ratio (0.669375 and 3.14, respectively). The F ratio was 9.8766 and *p*-value was 0.0067, which showed that the model is significant. The agitation rate was highly effective on the biosurfactant production where the F ratio and *p*-value were 8.9295 and 0.0092, respectively.

The activities of the biosurfactant produced after optimization were improved and the results showed that the surface tension of the produced surfactant was 35.8 mN/m, drop collapsing was 0.7 cm, oil displacement 4.5, and the emulsification index (E_24_) was 65.0% (Table 6).

### Time Course of Biosurfactant Production by *A. niger* SA1

**Fig. 3:**
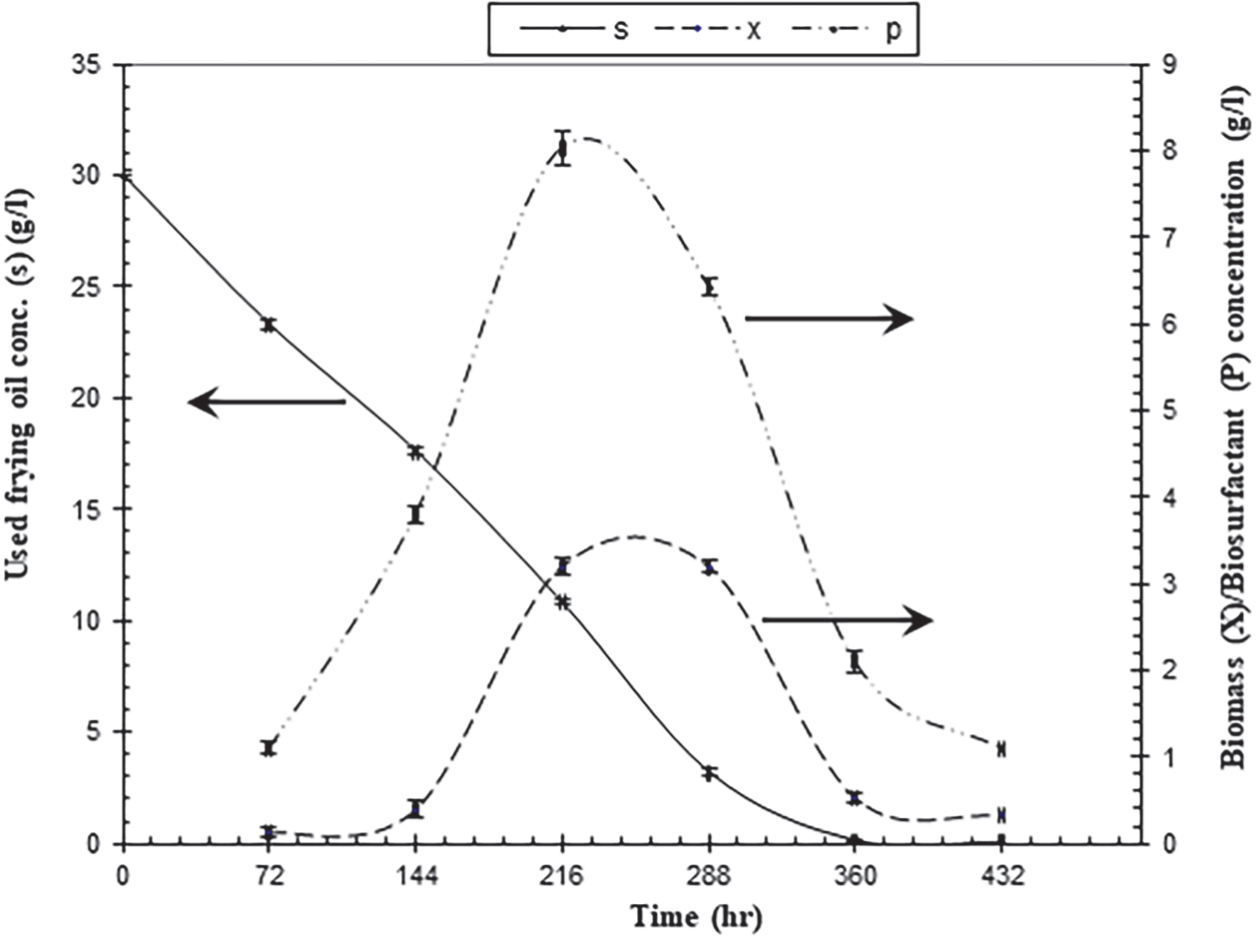
Effect of time course on the production of biosurfactant and fungal biomass of *A. niger* SA1 and the consumption rate of waste frying oil.

To perform a mathematical model to produce biosurfactant and biomass, the time course production was measured (Fig. 3, Table S4). The biosurfactant and biomass yield were increased with incubation time increased to 168 h (8.02 g/l and 3.2 g/l, respectively). A further increase in incubation time caused a decrease in biosurfactant and biomass production. This decrease was due to the consumption of waste frying oil (substrate). When comparing the production of biosurfactant by both *A. niger* SA1 and *A. niger* (type strain), which was isolated from non-oil-contaminated soil, strain SA1 produced (8.02 g/l), which was 2.4 fold more than the amount produced by the type strain (3.2 g/l) (Table S5).

### Mathematical Model of Biosurfactant and Biomass Production, and Oil Consumption

The mathematical model and kinetic parameters are summarized in Table 7. The main outputs of the model for biomass yield were Y_x/s_ (1.18), μ_max_ (0.0306), and Q_x_ (0.019); for biosurfactant yield they were Y_p/s_ (1.87), Y_p/x_ (2.51), and Q_p_ (0.048); where for waste frying oil consumption they were So (55), Q_s_ (0.26) and K_e_ (2.56).

By comparing the estimated data to the measured ones, the mathematical model was assessed; Figs. S4 and S5 showed the percentage errors between biomass and biosurfactant yields determined by experimental work and predicted using model equations at various stages of the fermentation process. The average error of biomass growth was 2.68%, as shown in Table 8, which showed the percentage error in *A. niger* SA1 biomass yield. To confirm the model’s accuracy, Fig. S4 depicted a comparison between the *A. niger* SA1 biomass yield that was calibrated and measured. It was discovered that this model adequately represented the increase of biomass. The percentage error in biosurfactant yield through fermentation is shown in Table 8, and the average error percentage was 3.39%.

## Discussion

A diversity of amphiphilic surface-active chemicals, known as biosurfactants, are created by living organisms, while hydrophobic fatty acids are connected to hydrophilic saccharides or peptides [40-42].

The soil sample (1) was the highest in fungal spores with 18 colonies, followed by soil sample (2) with 13 colonies and soil sample (10) with 5 colonies. *Aspergillus niger* was the most dominant fungal isolate on the two media (ECA and PCNB), followed by *A. terreus* and *A. egypticus*. The best-fitting substitution model for the alignment proved to be Kimura 2 parameter model with gamma distribution. A maximum-likelihood tree was constructed based on the ITS sequence of all strains with 1,000 bootstrap values given at the nodes. The spore density depends on the type and conditions of soil, so fungal spore density varies with different soils. These findings were similar to that found in a study in which exclosures had the highest overall spore density (60%) and stone terraces came in second (23%), while communal grazing fields had the lowest spore density (17%) [43, 44].

Similarly, *Aspergillus* was the most dominant and diverse genus in the soil studied [45]. Sixteen environmental isolates of *Aspergillus niger* were obtained from a variety of soils [46]. In the world of tiny filamentous fungi, *Aspergillus niger* is a universal representation [47].

All forty-four fungal isolates were capable of biosurfactant production except *Aspergillus nidulans*, *Chaetomium brasiliense*, *Chaetomium* sp., and *Emericella dentata*. The highest zone diameter was obtained from *A. niger*, followed by *A. flavus* and *Penicillium chrysogenium*. Comparatively, fungal biosurfactants make up only 19% of the total (12% come from ascomycetes and 7% come from basidiomycetes), but they have the widest range of chemical structural variations among all biosurfactants [48-50].

The production of biosurfactant from *A. niger* SA1 was the highest with surface tension, drop collapsing, oil displacement, and emulsification for the biosurfactant showing 35.8 mN/m, 0.55 cm, 6.7 cm, and 70%, respectively. The ability of *A. niger* to produce various types of substances makes it a very important species and garners attention for its potential industrial, medical, agricultural, environmental, and biotechnological applications [51-53]. As natural products that can be utilized in environmental applications or as additives to commercial products, biosurfactants are in high demand on the global market. Under many physicochemical circumstances, these biomolecules outperform chemical surfactants in terms of stability and the reduction of surface/interfacial tension between fluid phases. Biosurfactant manufacturing is still in its infancy in biotechnology [54].

Using a Fit Group model generated by JMP software to optimize the production of biosurfactant from *A. niger*, SA1, the biosurfactant yield was shown to be in the range of 2.39-8.02 g/l depending on the cultural conditions. The best culture conditions for the highest production of biosurfactant (8.02 g) were pH 6, temperature 35°C, waste frying oil 5.5 g, agitation rate 200 rpm, and an incubation period of 7 days. The lowest biosurfactant yield was obtained at the culture conditions of pH 7, temperature 25°C, waste frying oil 6.5 g, agitation rate 150 rpm, and an incubation period of 5 days.

Similar results have been obtained by many other researchers regarding the addition of oil as a substrate for biosurfactants and biomass production. The culture medium was supplemented with soybean oil at various concentrations, stimulating the growth of biomass [55]. In the presence of soybean oil, similar biomass values were seen for *Aspergillus* species, reaching maximum values of 4.49 g/l [56]. There have also been reports of a variety of oils being employed as culture medium substrates. Accordingly, biomass concentrations for sunflower oil, olive oil, and coconut oil were found to be 13.8 mg/ml, 15.2 mg/ml, and 13.1 mg/ml, respectively [57]. With leftover cooking olive oil, *Aspergillus niger* LFMB 1 produced higher biomass values (13.3 g/l) in 168 h [58].

Several patterns of biosurfactant generation via fermentation are feasible, depending on the type of biosurfactant and the generating microorganisms [59]. The biosurfactant and biomass yield were increased with incubation time increased to 168 h (8.02 g/l and 3.2 g/l, respectively) and a further increase in incubation time caused a decrease in biosurfactant and biomass production. This decrease was due to the consumption of waste frying oil (substrate).

After 3 days of incubation for *Mucor circinelloides* using the biosurfactant-producing medium, the highest yield of crude biosurfactant production (12.3 g/l) and the maximum growth rate (1.45 g/l dry cell weight) were attained [60]. It was discovered that the generation of rhamnolipids by *Pseudomonas aeruginosa*, emulsan by *Acinetobacter calcoaceticus* RAG-1, exopolysaccharide by *A. calcoaceticus* BD4, and surfactin by *Bacillus subtilis* in the culture broth were all related to growth [61-63]. *Rhodotorula babjevae* produced the most cell biomass (16.61 g/l) after 192 h of development, and the biosurfactant yield peaked at 72 h into the exponential growth phase [64]. The cultivation of *Candida lipolytica* in a medium supplemented with 6% soybean oil refinery residue and 1% glutamic acid led to a similar growth-associated generation of biosurfactant [65].

The activities of the biosurfactant produced after optimization were improved and the results showed that the surface tension of the produced surfactant was 35.8 mN/m, drop collapsing was 0.7 cm, oil displacement 4.5 cm, and the emulsification index (E_24_) was 65.0%. *A. niger* isolated from different sources such as from sugarcane bagasse was reported to produce biosurfactant with oil displacement of 0.133 cm and emulsification index of 48.067% [66]. Meanwhile, *A. niger* isolated from an industrial biotechnology laboratory showed 2.3 g/l with an emulsification index of 57% [67], and *A. niger* isolated from heavy metal-contaminated soil showed 5.6 g/1, oil displacement of 1.7 cm, and emulsification index of 61.3% [68].

Comparison of the estimated data with the measured data showed the average error of biomass growth to be 2.68%, which showed the percentage error in *A. niger* biomass yield. It was discovered that this model adequately represented the increase of biomass. The percentage error in biosurfactant yield through fermentation was calculated and the average error percentage was 3.39%.

It has also been demonstrated that statistical designs can be used to optimize bioprocess parameters, improving the manufacturing viability of these biomolecules, and in addition to reducing the number of tests performed, statistical experimental designs enable the verification of the influence of numerous factors, either separately or in relation to one another. The most important process variables can be identified using this technique, which then makes it possible to optimize the process [67, 69, 70].

A statistical design experiment called RSM involves simultaneously varying several elements. In fact, in an experiment, the correlation between the independent variables and the response variable is typically unknown. As a result, the first step is to roughly estimate the response variable by looking at the independent variables [71].

## Conclusion

As a result of using statistical and bioprocess tools, the biosurfactant yield was shown be 1.22 times higher than in nonoptimized conditions. The Fit Group statistical model was the best model for optimization while the logistic mathematical model was the most suitable model for prediction of the biosurfactant formation. The most effective factors in the production of biosurfactant were waste frying oil concentrations, agitation rate, temperature, and incubation time, while the least effective factor was pH value.

## Supplemental Materials

Supplementary data for this paper are available on-line only at http://jmb.or.kr.

## Figures and Tables

**Table 1:** Screening for biosurfactant production by isolated fungal isolates on CTAB-MB plates.

Code	Fungal strain	Soil no.	Zone diameter (mm)	Code	Fungal strain	Soil no.	Zone diameter (mm)
SA1	*Aspergillus niger*	1	7.60	SA23	*A. terreus*	9	5.75
SA2	*A. oryzae*	1	6.50	SA24	*A. egyptiacus*	2	5.55
SA3	*Aspergillus niger*	5	7.55	SA25	*A. egyptiacus*	2	5.58
SA4	*Aspergillus niger*	5	7.45	SA26	*A. nidulans*	3	ND
SA5	*Aspergillus niger*	5	7.50	SA27	*Chaetomium brasiliense*	4	ND
SA6	*Aspergillus niger*	5	7.57	SA28	*Chaetomium brasiliense*	4	ND
SA7	*Aspergillus niger*	6	7.24	SA29	*Aspergillus niger*	1	6.54
SA8	*Aspergillus niger*	6	7.23	SA30	*Aspergillus niger*	1	6.48
SA9	*Aspergillus niger*	6	7.25	SA31	*Aspergillus niger*	2	6.55
SA10	*Aspergillus niger*	6	7.24	SA32	*Aspergillus niger*	2	6.65
SA11	*Penicillium chrysogenum*	6	5.90	SA33	*Aspergillus niger*	2	6.36
SA12	*Penicillium chrysogenum*	6	5.95	SA34	*Aspergillus niger*	5	6.66
SA13	*Penicillium chrysogenum*	6	5.97	SA35	*Aspergillus niger*	5	6.65
SA14	*Penicillium chrysogenum*	6	5.94	SA36	*Emericella dentata*	7	ND
SA15	*A. terreus*	1	5.80	SA38	*Chaetomium* sp.	7	ND
SA16	*A. terreus*	1	5.86	SA39	*A. terreus*	10	5.55
SA17	*A. terreus*	1	5.84	SA40	*A. terreus*	10	5.66
SA18	*A. terreus*	1	5.80	SA41	*A. egyptiacus*	9	5.35
SA19	*A. terreus*	1	5.88	SA42	*A. egyptiacus*	9	5.42
SA20	*Aspergillus niger*	1	7.55	SA43	*A. egyptiacus*	10	5.33
SA21	*Aspergillus niger*	1	7.56	SA44	*A. egyptiacus*	10	5.35
SA22	*A. terreus*	9	5.57				

ND: not detectable

**Table 2:** Production of biosurfactant and surface activity of the selected fungal isolates.

Code	Fungal strain	Biosurfactant (g l^-1^)	Surface activity			
			Surface tension (mNm^-1^)	Drop collapsing (cm)	Oil displacement (cm)	Emulsification index (%)
SA1	*Aspergillus niger*	6.6 ± 0.20	35.5 ± 1.6	0.55 ± 0.04	6.7 ± 0.33	70 ± 2.2
SA2	*A. oryzae*	5.1 ± 0.27	50.3 ± 1.8	0.45 ± 0.05	5.0 ± 0.41	65 ± 2.8
SA13	*Penicillium chrysogenum*	4.7 ± 0.42	35.4 ± 1.2	0.40 ± 0.06	5.5 ± 0.35	50 ± 3.1
SA19	*A. terreus*	5.4 ± 0.28	52.5 ± 1.6	0.48 ± 0.05	4.5 ± 0.38	67 ± 2.4
SA25	*A. egyptiacus*	4.9 ± 0.27	52.1 ± 1.1	0.45 ± 0.05	5.0 ± 0.42	59 ± 2.5

**Table 3:** Experimental results of the Fit Group model for optimization of biosurfactant production generated by JMP statistical software.

Run	X1 pH	X2 Temperature (°C)	X3 Waste frying oil (g)	X4 Agitation rate (rpm)	X5 Incubation time (day)	Y Biosurfactant (g)
1	6	30	6.5	200	7	5.93
2	5	35	6.5	150	5	6.07
3	7	35	6.5	150	9	4.56
4	5	35	4.5	150	9	5.85
5	6	35	5.5	200	7	8.02
6	7	35	4.5	150	5	5.50
7	5	35	4.5	250	5	6.28
8	5	25	6.5	250	5	5.37
9	7	25	6.5	150	5	2.39
10	7	35	4.5	250	9	2.53
11	7	25	4.5	150	9	5.25
12	7	25	4.5	250	5	2.93
13	5	30	5.5	200	7	5.05
14	7	25	6.5	250	9	4.50
15	6	30	5.5	200	7	4.47
16	5	25	4.5	250	9	4.41
17	5	25	4.5	150	5	5.88
18	6	30	5.5	250	7	3.84
19	6	30	5.5	200	5	4.48
20	7	35	6.5	250	5	6.45
21	5	25	6.5	150	9	3.45
23	5	35	6.5	250	9	4.53

**Table 4:** Final parameter estimates for the remaining terms after model selection.

Term	Estimate	Std Error	Lower 95%	Upper 95%	t Ratio	Prob>|t|
Intercept	5.0552	0.405621	4.19064	5.91976	12.46	<0.0001*
X1 (5,7)	-0.4544	0.208007	-0.8978	-0.011	-2.18	0.0452*
X2 (25,35)	0.52095	0.210177	0.07297	0.96893	2.48	0.0256*
X5*X4	0.669375	0.212994	0.21539	1.12336	3.14	0.0067*
X3*X5	-0.491875	0.22994	-0.9459	-0.0379	-2.31	0.0356*
X4*X4	-1.9604	0.656043	-3.3587	-0.5621	-2.99	0.0092*
X5*X5	1.69865	0.653257	0.30626	3.09104	2.60	0.0201*

The following terms were excluded from the final model: X2(4.5,6.5), X3(5,9), X4(150,250), X1*X2, X1*X3, X2*X3, X1*X4, X3*X4, X1*X5, X2*X5, X4*X5, X1*X1, X2*X2, X3*X3.

**Table 5:** Analysis of variance (ANOVA) for the remaining sources after model selection.

Source	Nparm	DF	Sum of Squares	F Ratio	Prob>F
X1 (5,7)	1	1	3.4639629	4.7722	0.0452*
X5 (25,35)	1	1	4.4593907	6.1436	0.0256*
X2*X4	1	1	7.1690063	9.8766	0.0067*
X3*X5	1	1	3.8710563	5.3331	0.0356*
X4*X4	1	1	6.4815336	8.9295	0.0092*
X5*X5	1	1	4.9078585	6.7614	0.0201*

**Table 6:** Surface activity of crude biosurfactant produced by *A. niger* (SA1) after optimization.

Parameter	Measurements
Surface tension (mNm^-1^)	35.8 ± 1.8
Drop collapsing (cm)	0.7 ± 0.03
Oil displacement (cm)	4.5 ± 0.31
Emulsification index E_24_ (%)	65.0 ± 4.5

**Table 7:** The mathematical model obtained by regression of biosurfactant and biomass production and waste frying oil consumption by *A. niger* (SA1) further the fermentation process.

Parameters		Measurements
Biomass yield	X_o_ (g l^-1^)	0.08316
	X_max_ (g dry wt. l^-1^)	3.5
	Y_x/s_ (g dry wt. g^-1^)	1.1799
	μ_max_ (h^-1^)	0.0306
	Q_x_ (g dry wt. h^-1^)	0.019
	q_x_ (g dry wt. g^-1^ oil consumed h^-1^)	0.007
Biosurfactant yield	P_o_ (g l^-1^)	1.4
	Y_p/s_ (g g^-1^ oil consumed)	1.8736
	Y_p/x_ (g g^-1^ dry wt.)	2.5094
	Q_p_ (g l^-1^ h^-1^)	0.0478
	q_p_ (g g^-1^ dry wt. h^-1^)	0.0149
	A	1.5371
	K	1.2989
Waste frying oil consumption	S_o_ (g l^-1^)	55
	Q_s_ (g oil consumed l^-1^ h^-1^)	0.2631
	q_s_ (g oil consumed g^-1^ dry wt. h^-1^)	0.0822
	K_e_	2.560

Note: X_o_: initial biomass concentration, X_max_: maximum biomass yield, Y_x/s_: biomass yield coefficient, μ_max_: maximum specific growth rate, Q_x_: biomass production rate, q_x_: specific biomass yield rate, P_o_: initial biosurfactant formed, Y_p/s_: biosurfactant yield coefficient, Y_p/x_: specific biosurfactant yield coefficient, Q_p_: biosurfactant formation rate, q_p_: specific biosurfactant formation rate, α: biosurfactant yield coefficient, K: constant of integration, S_o_: initial oil consumed, Q_s_: oil consumed rate, q_s_: specific oil consumed rate, Ke: maintenance coefficient.

**Table 8:** Comparison between experimental and calculated data for biosurfactant and biomass yields.

Time (t)	Biomass yield			Biosurfactant yield		
	X_experimental_	X_calculated_	Error %	P_experimental_	P_calculated_	Error %
72	0.05	0.045	10.00	1.1	1.13	-2.73
120	0.25	0.27	-8.00	3.8	3.85	-1.32
168	3.2	3.1	3.13	8.02	7.9	1.56
216	3.2	3	6.25	6.42	6.2	3.43
264	2.3	2.1	8.70	2.1	1.98	5.71
