# Supplementary material for: Statistical Optimization of Biosurfactant Production from Aspergillus niger SA1 Fermentation Process and Mathematical Modeling
Source: J Microbiol Biotechnol. 2023 Jun 9;33(9):1238–49. doi: 10.4014/jmb.2303.03005 (PMC10580895; doi:10.4014/jmb.2303.03005)
Supplement: Supplementary file 1 [file jmb-33-9-1238-supple.pdf]

J. Microbiol. Biotechnol.
https://doi.org/10.4014/jmb.2303.03005

Supplementary Tables and Figures

Table S1. Coordinates of the sampling sites.

Sampling site	Coordinates	
	N	E
Khulais 1	22° 08′ 54.46"	39° 20′ 35.02"
Khulais 2	22° 06′ 35.90"	39° 18′ 41.80"
Khulais 3	22° 27′ 57.80	39° 24 ′ 16.80
Khulais 4	22° 22 ′ 69.71	39°.25 ′ 79.81
Mecca old road 1	21° 27′ 07.30"	39° 36′ 09.70"
Mecca old road 2	21° 25′ 59.44"	39° 38′ 28.75"
Mecca old road 3	21° 24′ 57.31"	39° 40′ 41.30"
Asfan road 1	21° 54′ 56.87"	39° 20′ 09.80"
Asfan road 2	21° 51′ 57.70"	39° 24′ 58.07"
Asfan road 3	21° 48′ 06.76"	39° 28′ 52.57"

Table S2a. Summarization of the factors used to build up the model.

Factors	Role	Changes	Values
X1 (pH)	Discrete Numeric	Easy to change	5, 6, 7
X2 (Temperature °C)	Discrete Numeric	Easy to change	25, 30, 35
X3 (Waste frying oil g)	Discrete Numeric	Easy to change	4.5, 5.5, 6.5
X4 (agitation rat rpm)	Discrete Numeric	Easy to change	150, 200, 250
X5 (incubation time days)	Discrete Numeric	Easy to change	5, 7, 9

Table S2b. Responses

Response(s)	Goal	Limits	Importance	Detection Limits
Y	Maximize	NA	NA	NA

Table S3. Effect summary of the different media compositions (factors) and their combinations.

Source	FDR Log worth	FDR P Value
X2*X4	2.174	0.00671
X4*X4	2.037	0.00919
X5*X5	1.697	0.02009
X2 (25,35)	1.592	0.02556
X3*X5	1.449	0.03558
X1(5,7)	1.345	0.04521

X1, pH value; X2, Temperature; X3, Waste frying oil; X4, agitation rate; X5, incubation time.

Table S4. Time course of biomass yield (g dry wt.), substrate consumption (g), and biosurfactant produced (g) by *A. niger* SA1 on optimized medium.

Time (day)	Time (hr.)	Biomass yield (g dry wt. l^{-1})	Substrate residual (g l^{-1})	Biosurfactant (g l^{-1})
3	72	0.13±0.02	23.3±0.20	1.10±0.01
5	120	0.39±0.02	17.6±0.21	3.80±0.07
7	168	3.20±0.10	10.8±0.15	8.02±0.09
9	216	3.20±0.10	3.20±0.11	6.42±0.10
11	264	0.53±0.03	0.12±0.10	2.10±0.08
13	312	0.32±0.03	0.04±0.09	1.10±0.09

Table S5. Comparison of the production of biosurfactant from the *Aspergillus niger* SA1 isolated from the oil-contaminated soil and *Aspergillus niger* isolated from non-oil-contaminated soil as a control.

Time	Time	Biosurfactant (g l^{-1})	
(day)	(hr.)	*A. niger* SA1	*A. niger* (type strain)
3	72	1.10±0.01	0.7±0.05
5	120	3.80±0.07	1.3±0.70
7	168	8.02±0.09	3.3±0.40
9	216	6.42±0.10	1.2±0.10
11	264	2.10±0.08	0.9±0.01
13	312	1.10±0.09	0.5±0.01

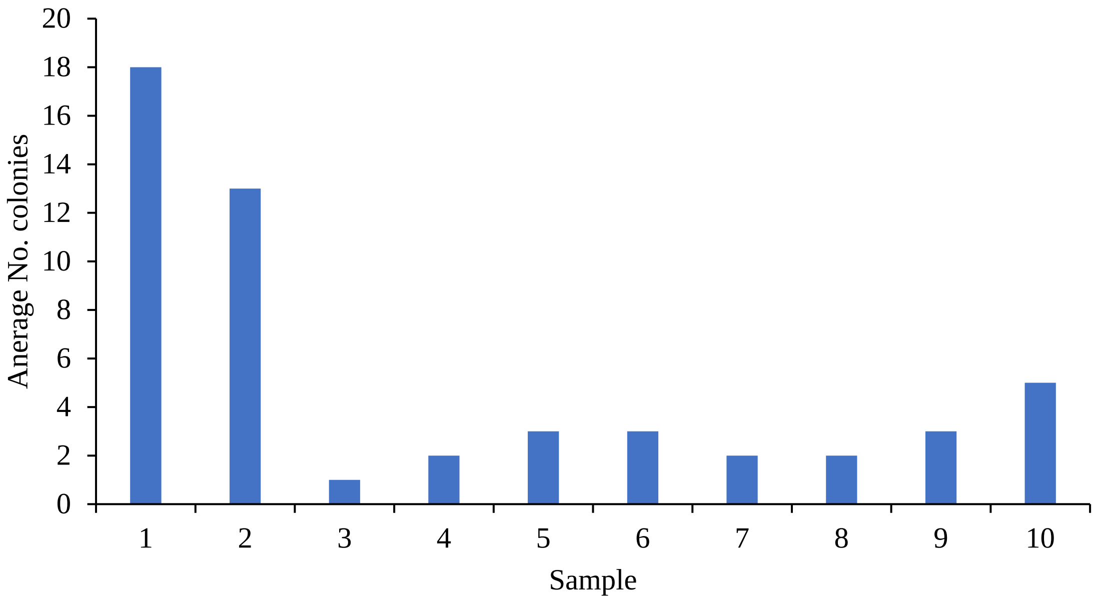

Fig. S1. Spore densities in the 10 Saudi Arabian soil samples collected from petroleum oil-polluted areas.

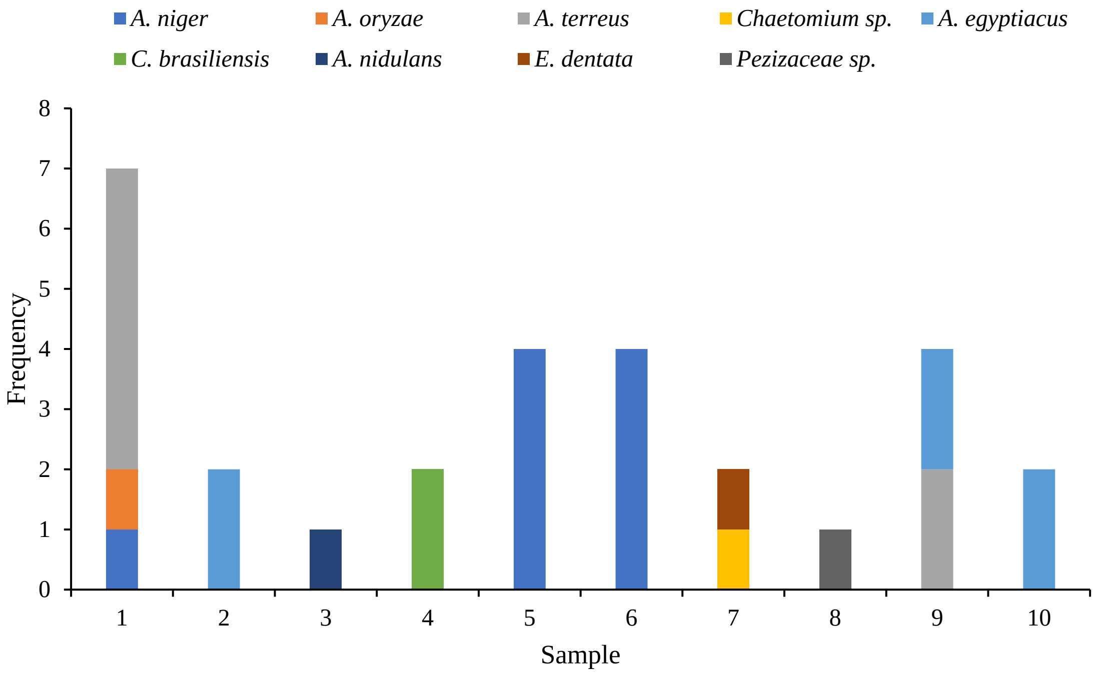

Fig. S2. Numbers of isolates per species and soil sample, isolated on ECA medium.

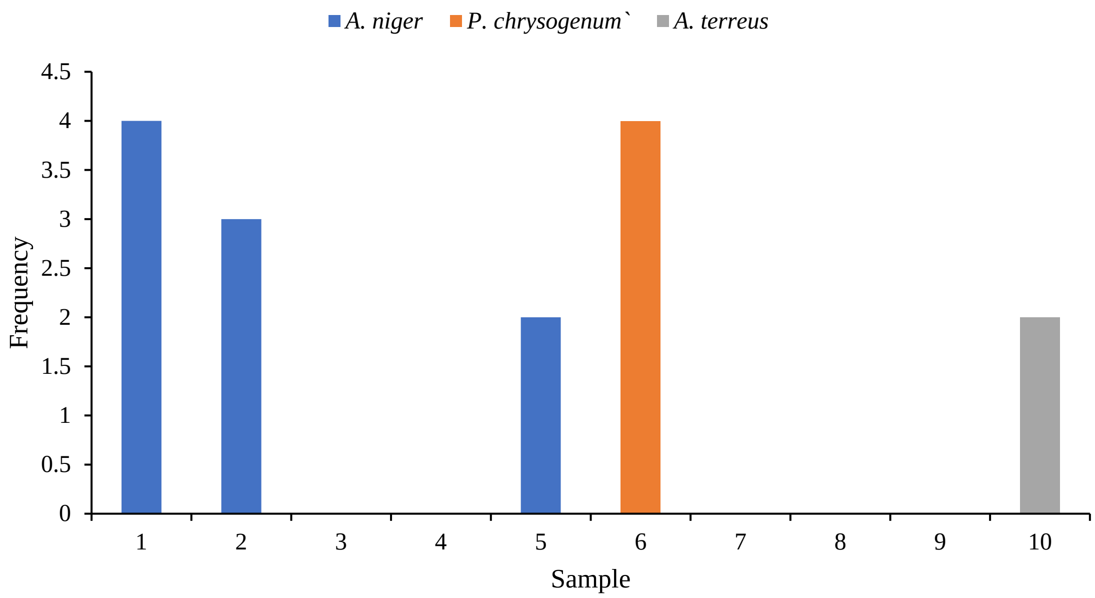

Fig. S3. Numbers of isolates per species and soil sample, isolated on PCNB medium.

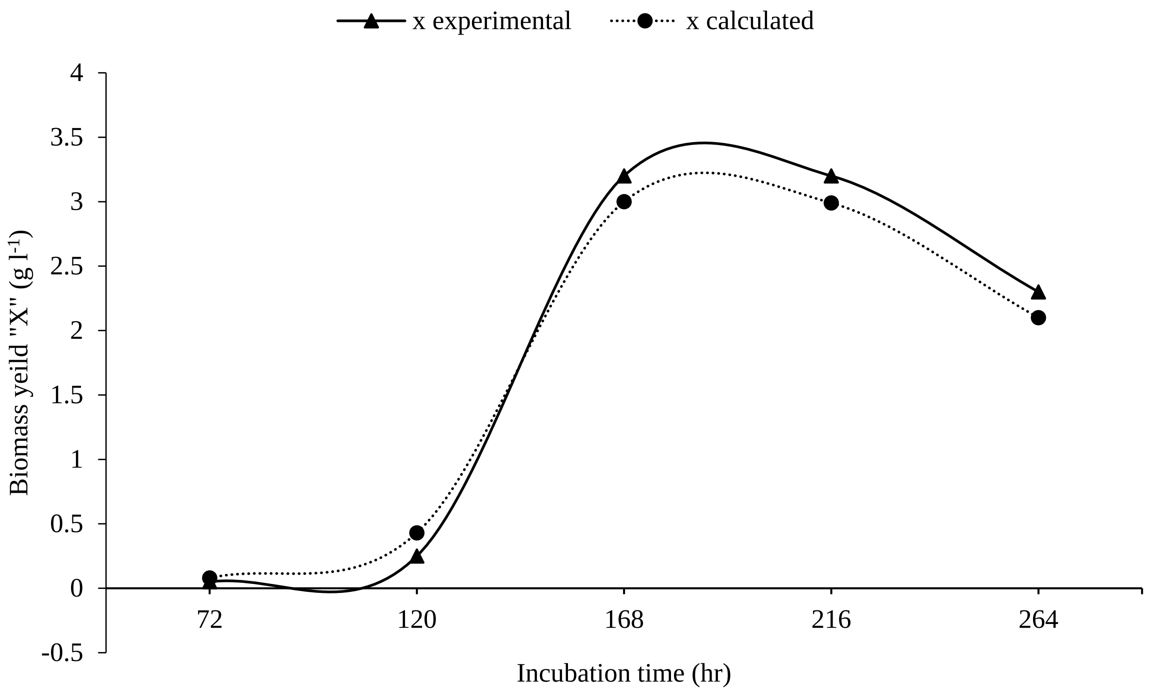

Fig. S4. Comparison between the experimental yield of biomass of *A. niger* SA1 and the calculated biomass yield using the mathematical model.

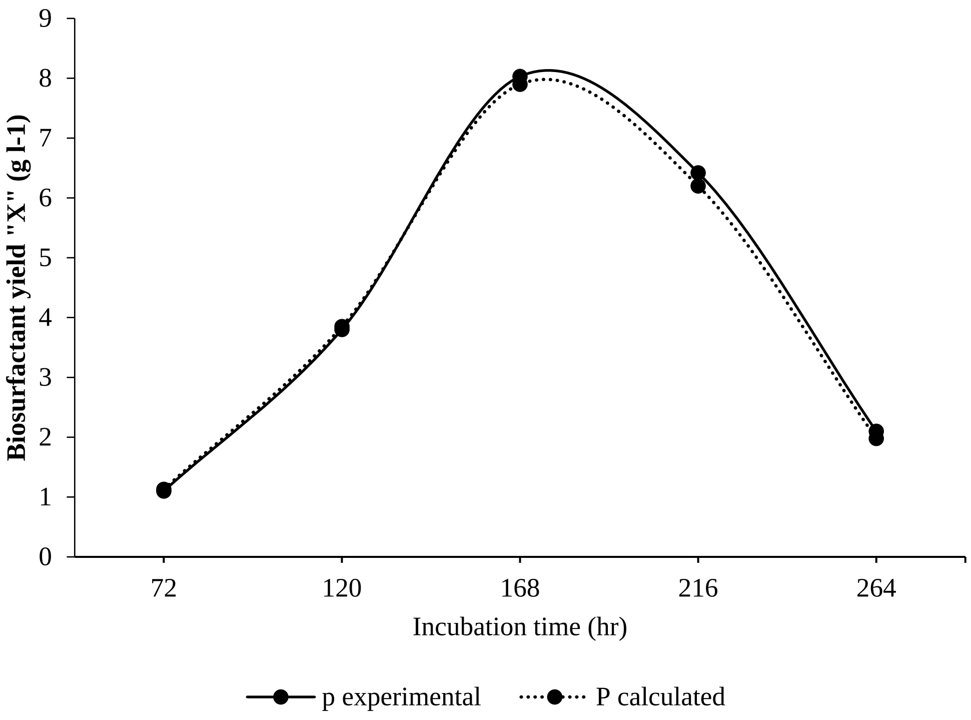

Fig. S5. Comparison between the experimental yield of biosurfactant by *A. niger* SA1 and the calculated biosurfactant yield using the mathematical model.
